# Competitive and Cooperative CO_2_–H_2_O Adsorption through Humidity Control in a Polyimide Covalent Organic Framework

**DOI:** 10.1021/acsami.3c04561

**Published:** 2023-06-09

**Authors:** Hugo Veldhuizen, Saira Alam Butt, Annemiek van Leuken, Bart van der Linden, Willy Rook, Sybrand van der Zwaag, Monique A. van der Veen

**Affiliations:** † Department of Novel Aerospace Materials, Delft University of Technology, Delft 2629 HS, The Netherlands; ‡ Department of Catalysis Engineering, Delft University of Technology, Delft 2629 HZ, The Netherlands

**Keywords:** covalent organic frameworks, CO_2_ capture, relative humidity, cooperative adsorption, breakthrough experiments, FT-IR spectroscopy

## Abstract

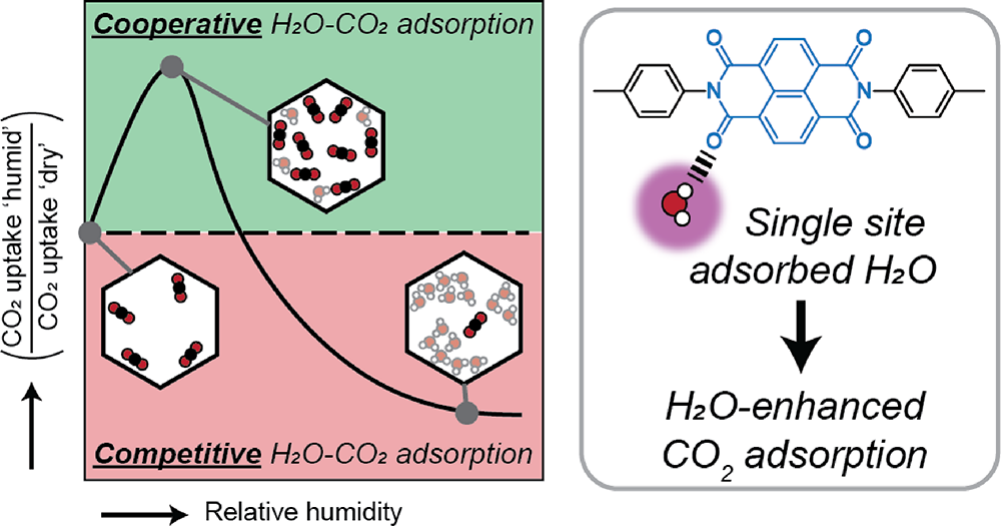

In order to capture and separate CO_2_ from the air or flue gas streams through nanoporous adsorbents, the influence of the humidity in these streams has to be taken into account as it hampers the capture process in two main ways: (1) water preferentially binds to CO_2_ adsorption sites and lowers the overall capacity, and (2) water causes hydrolytic degradation and pore collapse of the porous framework. Here, we have used a water-stable polyimide covalent organic framework (COF) in N_2_/CO_2_/H_2_O breakthrough studies and assessed its performance under varying levels of relative humidity (RH). We discovered that at limited relative humidity, the competitive binding of H_2_O over CO_2_ is replaced by cooperative adsorption. For some conditions, the CO_2_ capacity was significantly higher under humid versus dry conditions (*e.g.*, a 25% capacity increase at 343 K and 10% RH). These results in combination with FT-IR studies on equilibrated COFs at controlled RH values allowed us to assign the effect of cooperative adsorption to CO_2_ being adsorbed on single-site adsorbed water. Additionally, once water cluster formation sets in, loss of CO_2_ capacity is inevitable. Finally, the polyimide COF used in this research retained performance after a total exposure time of >75 h and temperatures up to 403 K. This research provides insight in how cooperative CO_2_–H_2_O can be achieved and as such provides directions for the development of CO_2_ physisorbents that can function in humid streams.

## 1 Introduction

Physical adsorption of CO_2_ on solid adsorbents has been demonstrated to be a promising upcoming technology for CO_2_ removal from flue gas. It distinguishes itself form traditional processes such as amine absorption, by being a process with cost- and energy-efficient regeneration of the adsorbent.^1^ In addition, the non-corrosive nature of the physical adsorbents, makes storage and maintenance less complicated. Activated carbons, zeolites, metal–organic frameworks (MOFs), and covalent organic frameworks (COFs) have been widely studied as nanoporous physical adsorbents for carbon capture under experimental conditions similar to those in industrial applications (*i.e.*, multi-component gas separation in a packed bed).^2^ MOFs and COFs, in particular, are promising materials since their frameworks are highly tunable through the vast library of building blocks that can be used to construct them. In this way, MOFs and COFs have been molecularly engineered to display both a high CO_2_ capacity and framework robustness.^3−6^ Depending on the chemical structure and topology, subclasses of these materials retain a high selectivity toward CO_2_ adsorption when they are exposed to mixed gas streams of N_2_, H_2_, and/or CH_4_. The most used experimental setup to verify their potential for post-combustion carbon capture concerns exposing a packed bed of adsorbent to a mixed stream of N_2_/CO_2_ of ratios ranging from 80/20 to 95/5, as these represent typical N_2_/CO_2_ ratios in flue gas.^4,7^

Water vapor, an ubiquitous component in flue gas, often lowers the efficiency of these adsorbents through competitive adsorption of water over CO_2_.^8^ This phenomenon depends first on the concentration-dependent affinities (*i.e.*, isosteric enthalpy of adsorption) of CO_2_ and H_2_O towards the adsorbent. Water–adsorbent enthalpies are often higher and adsorbed water provides new adsorption sites for multilayer water sorption (*i.e.*, water clusters), which results in complete pore filling at high relative humidity (RH) values. Although CO_2_ sorption is often diminished in the presence of water,^6^ in some cases, it is unaffected or even enhanced, depending on the RH and specific adsorbent.^9,10^ Recent examples of unaffected CO_2_ adsorption in humid conditions are from the group of Smit and co-workers.^3^ In their study, they computationally screened a library of 300,000 MOFs to discover specific structural motifs that enables – once implemented in a framework – high CO_2_/N_2_ selectivity, which persists in wet flue gases. In their study, they discovered (*in silico*) parallel aromatic motifs with a distance of 7 Å as a highly selective CO_2_ binding site. Once this segment was experimentally included in a hydrophobic MOF, CO_2_ adsorption performance was retained in breakthrough experiments with humidified streams using 85% RH in the feed flow, throughout multiple consecutive cycles. However, initially dry adsorbents are used in these experiments as well as desorption steps at higher temperatures. As a result of such an experimental procedure and the inherent slow diffusion of water in nanoporous materials,^11^ an overall lower RH level is likely present in the adsorption column. In another study regarding a novel zinc triazolate oxalate framework, a rare phenomenon of competitive binding of CO_2_ over H_2_O was observed.^4^ There, as the result of the specific ultramicroporous architecture, water cluster formation is sterically hindered: the adsorption of CO_2_ causes the breaking of H-bonds in the water cluster, while due to space limitations, no alternative H-bonds can be formed. This means that until 40% RH, CO_2_ (at 1 bar) is competitively adsorbed, such that it even leads to water expulsion.

Enhanced CO_2_ sorption in the presence of water also occurs with specific adsorbents that contain small mesopores at controlled RH values, for example, in the MOF MIL-100(Fe) bearing 2.5 and 2.9 nm mesoporous cages.^10,12^ Pre-equilibrated water is able to effectively bring the pore walls closer together and subsequently form microporous pockets, which causes the retention of a higher concentration of CO_2_ than in the dry state. On the other hand in microporous PCN-250 MOFs, CO_2_ adsorption capacities can be enhanced 1.7 times when adsorption is being executed with humified gas streams compared to dry streams.^13^ This particular improvement is said to be due to H_2_O molecules clamping the CO_2_ molecules on the open metal sites, which uses the adsorption sites more effectively than in the dry situation. Also compared to dry conditions, relative humidity values of 20% are causing 1.5- and 2.4-fold CO_2_ capacity increases in MOFs MIL-53(Al) and NOTT-400, respectively.^14,15^ In these cases, the bridging hydroxo-groups within the MOF are providing water adsorption sites. In the case of MIL-101, water can coordinate to the otherwise exposed Cr-sites, and these terminal water molecules act as additional interaction sites to enhance CO_2_ uptake, particularly at low pressures.^16^ Pre-adsorbed water at low RH values provide more favorable CO_2_ binding sites than the dry variants of these structures. Although COFs seem to be equally suited for CO_2_ separation from humid gas streams, there is a lack of detailed COF studies with systematic RH variations to investigate potentially similar water-enhanced effects as have been observed for MOFs. Among few of these examples in the COF field,^17^ there are fewer experimental studies that attempt to relate COF chemistry and structure to CO_2_ separation performance under humid environments as encountered in industrially relevant setups. An extensive study involving imine-COFs assessed the performance of NUS-2 and TpPa-1 (promising COFs in terms of high CO_2_ capacity and stability) in N_2_/CO_2_ breakthrough experiments where the adsorbents are pre-saturated with water.^6^ This study showed that at 17% RH, NUS-2 and TpPa-1 retained about 70% of their dry CO_2_ adsorption capacities over prolonged periods of time. Nevertheless, CO_2_ breakthrough studies in the presence of water using COF adsorbents always report a negative effect of water.^6,7^ Expanding on these studies with different COF structures at various RH values would allow for a fair assessment of the potential of COFs for industrial CO_2_ separation from humid streams. In addition, these studies unravel the possibility of water-enhanced CO_2_ adsorption as has been found in specific MOFs.

Here, we systematically controlled the RH values to which the adsorbent is exposed in N_2_/CO_2_/H_2_O breakthrough studies, to assess the material and experimental requirements for control over the competitive or cooperative binding of CO_2_ and H_2_O. We chose a polyimide COF as a model COF system for this particular study, synthesized from 1,3,5-tris(4-aminophenyl)benzene (TAPB) and 1,4,5,8-naphthalenetetracarboxylic dianhydride (NDA), the reason being the overall hydrolytic and mechanical stability of polyimide COFs, the relative ease of preparation, and their commercially available building blocks. Furthermore, the TAPB-NDA COF contains a significant supermicropore and mesopore volume, which seems to be beneficial in achieving water-assisted CO_2_ adsorption. The large degree of aromatic planes could potentially also promote the presence of the aromatic motifs that Smit *et al.* identified as capable of competitively adsorbing CO_2_ over water.

## 2 Results and Discussion

**Figure 1:**
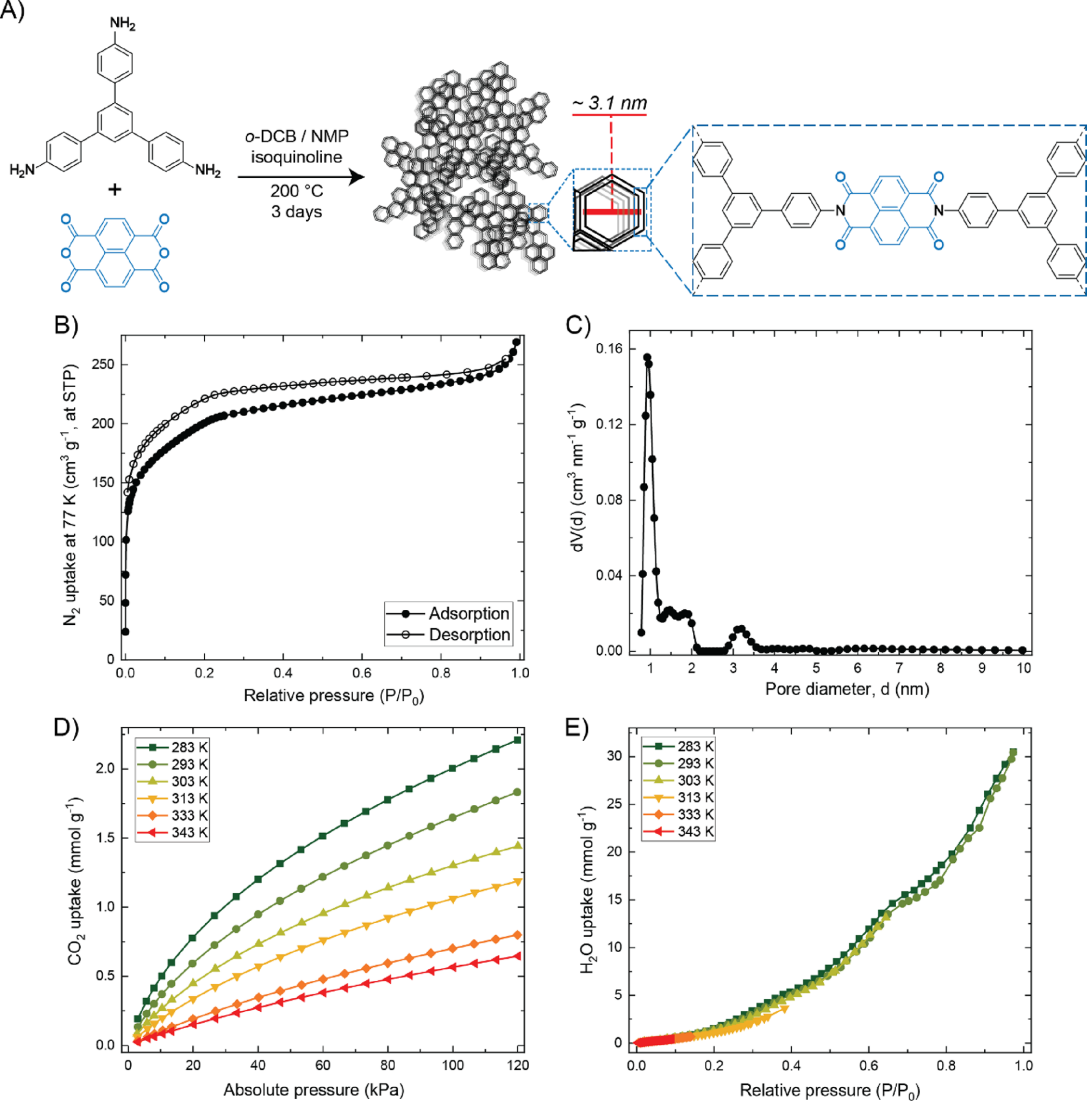
(A) TAPB-NDA-COF synthesis conditions and representation of its chemical and porous structure. (B) Nitrogen sorption isotherms of TAPB-NDA-COF at 77 K. (C) Pore size distribution based on the adsorption branch of the nitrogen isotherm and a QSDFT carbon model. D) CO_2_ adsorption isotherms of TAPB-NDA-COF at 283, 293, 303, 313, 333, and 343 K. (E) H_2_O vapor adsorption isotherms (relative pressure) of TAPB-NDA-COF at 283, 293, 303, 313, 333, and 343 K.

The solvothermal polycondensation of 1,3,5-tris(4-aminophenyl)benzene (TAPB) and 1,4,5,8-naphthalenetetracarboxylic dianhydride (NDA) was executed in a sealed glass flat-bottom 100 mL cylindrical reactor and yielded the nanoporous polyimide polymer named TAPB-NDA-COF (Figure 1A). The powder was then subjected to pelletization (hydraulic press, 30 MPa) and sieving (fractions of 300–425 μm), after which these COF pellets were characterized. The completion of the polymerization was confirmed by FT-IR analysis (Figure S1) and the TGA profile of the TAPB-NDA-COF (Figure S2), revealing a 5% weight loss temperature of 535 °C at a heating rate of 10 °C·min^–1^ under a N_2_ atmosphere, which is expected behavior for polyimides confirming their thermal stability. The nanoporous polymer network was further characterized by PXRD and N_2_ sorption. The former technique was used to classify this TAPB-NDA-COF as semi-crystalline since there are noticeable reflections at (100) (from 2.8° 2θ, corresponding to the expected hexagonal size of 3.1 nm) and (200) but no peaks indicative of long-range order (Figure S3). Lastly, the nitrogen sorption isotherms provided insights into the porous architecture of the COF (Figure 1B). The BET surface area of the TAPB-NDA-COF calculated from the adsorption isotherm is 722 m^2^·g^–1^, of which analysis details are provided in the Supporting Information (Figure S4). The adsorption branch shows a steep N_2_ uptake until 0.02 *P*/*P*_0_ and a more gradual N_2_ uptake between 0.02 and 0.3 *P*/*P*_0_, after which the curve plateaus. Such features are indicative of a distribution of micro- and mesopores being present in the framework, which prompted us to calculate a pore size distribution (PSD) based on the experimental data of this adsorption branch (further specified in Supporting Information, Figures S5 and S6). The PSD of the TAPB-NDA-COF is depicted in Figure 1C, showing distinct supermicropore volume (1–2 nm, ∼0.27 cm^3^·g^–1^) and a relatively small mesopore volume (at 3.1 nm, ∼0.03 cm^3^·g^–1^). Thus, the accessible pore volume originates not exclusively from the crystallographic unit of 3.1 nm based on molecular simulations.^18^ This prominent supermicropore volume plays an important role in the CO_2_ capacity of the COF.

The functional gas sorption properties of the COF were first studied in a controlled, single-component environment in order to later compare the capacities with values obtained from breakthrough experiments. The CO_2_ uptake at 1 bar varied from 2.0 to 0.5 mmol·g^–1^ at adsorption temperatures from 283 to 343 K (Figure 1D).^19^ In addition, serving as benchmark values for the experimental data of the breakthrough studies using 20/80 CO_2_/N_2_ mixtures at 3 bar, the CO_2_ uptake at 0.6 bar varied from 1.5 to 0.3 mmol·g^–1^ at adsorption temperatures from 283 to 343 K. Although seemingly the absolute H_2_O uptake drops at higher adsorption temperatures, plotting the water uptake as a function of relative pressure (*i.e.*, relative humidity) shows overlapping isotherms for all temperatures (Figure 1E). The COF’s water vapor capacity at RH 90% of 30 mmol·g^–1^ (> 50 wt %) is comparatively high.^20^ The small yet significant water uptake even at low RH values (*e.g.*, 1.5 mmol·g^–1^ at 20% RH) suggests some hydrophilicity within the COF. The polymer backbone is largely structured by hydrophobic benzene rings, but hydrophilicity may originate from the imide bonds, where the high electron density around the oxygen atoms can result in hydrogen bonding with water. Finally, the isosteric enthalpy of adsorption (Δ*H*_ads_) of CO_2_ is calculated based on its isotherms at multiple temperatures. The Δ*H*_ads_ of CO_2_ adsorbed on TAPB-NDA-COF varies from −35 to −28 kJ·mol^–1^ at 0.1 and 0.8 mmol·g^–1^ loading, respectively (Figure S7).

**Figure 2:**
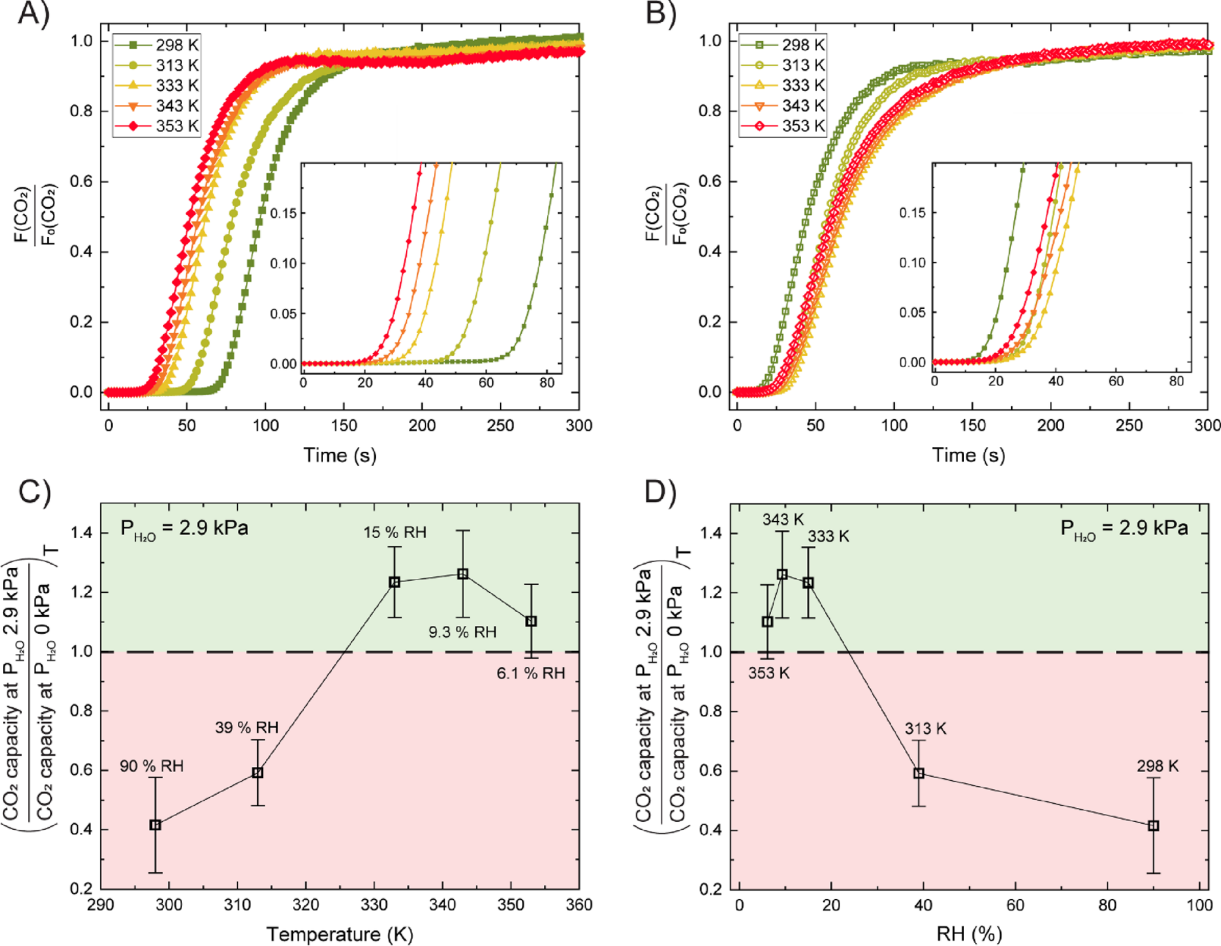
(A) CO_2_ breakthrough curves from dry 20/80 CO_2_/N_2_ mixed gas over a packed bed of TAPB-NDA-COF at 298, 313, 333, 343, or 353 K, all at 3.1 bar total pressure (all cycle 2). (B) CO_2_ breakthrough curves from humid (*P*(H_2_O) = 2.9 kPa) 20/80 CO_2_/N_2_ mixed gas over a packed bed of TAPB-NDA-COF at 298, 313, 333, 343, or 353 K, all at 3.1 bar (all cycle 2). (C) Ratio of the CO_2_ capacity at *P*(H_2_O) = 2.9 kPa over the CO_2_ capacity at *P*(H_2_O) = 0 kPa, plotted as a function of temperature. Error bars are calculated based on 3 to 4 consecutive breakthrough cycles. (D) The same data as panel (C), plotted now as a function of relative humidity.

A series of breakthrough studies on TAPB-NDA-COF was executed to assess the COF’s CO_2_ separation performance under dry and humid conditions. The COF was always first equilibrated with a humid helium stream at the same temperature and water vapor pressure as that of the ensuing CO_2_/N_2_ breakthrough experiment. 20/80 CO_2_/N_2_ feed mixtures were used at 3.1 bar and various temperatures: 298, 313, 333, 343, and 353 K (for detailed experimental methods, see Supporting Information, Figures S8–S12). For the humid streams, the inlet stream was humidified through a saturator at room temperature leading to a constant partial water vapor pressure of 2.9 kPa. The temperature of the column would then determine the effective relative humidity. Thus, RH values of 6, 9, 15, 39, and 90% were imposed for column temperatures of 353, 343, 333, 313, and 298 K, respectively. Figure 2A,B displays the CO_2_ breakthrough curves (as a ratio of estimated exit flow rate over the feed flow rate) of these experiments for dry and humid gas streams, respectively. In both cases, *t* = 0 s represents the first detection of non-adsorbing N_2_ gas. The breakthrough time and curve slope dictate the CO_2_ capacity of the adsorbent as it can be quantified as the area above the curve. Each experiment contained 3 to 4 consecutive cycles of adsorption and desorption, of which the CO_2_ breakthrough times and capacities have been calculated (Table 1). In the case of dry gas streams, CO_2_ has shorter breakthrough times and smaller CO_2_ adsorption capacity as the temperature increases (see Table 1), in line with the expectations based on the CO_2_ adsorption isotherms at different temperatures. Inversely, for the humid CO_2_/N_2_ gas streams, CO_2_ breakthrough times and CO_2_ adsorption capacity (see Table 1) initially increase with temperature, with a maximum at 343 K. Under humid conditions, the CO_2_ breakthrough curves become less steep at higher temperatures, indicative of more dispersion.

**Table 1:** Resulting CO_2_ Breakthrough Times and CO_2_ Capacities from Experiments of 20/80 CO_2_/N_2_ Mixed Gas over a Packed Bed of TAPB-NDA-COF at 298, 313, 333, 343, or 353 K, All at 3.1 bar, Either in Dry or Humid Conditions (*P*(H_2_O) = 2.9 kPa)^a^

adsorption temperature (K)	P(H_2_O) (kPa) dry // humid	CO_2_ breakthrough time (s) dry // humid	CO_2_ capacity (mmol·g^–1^) dry // humid
298	0 // 2.9	63 ± 1.0 // 9.1 ± 1.5	1.3 ± 0.029 // 0.56 ± 0.20
313	0 // 2.9	45 ± 3.6 // 21 ± 2.7	1.1 ± 0.052 // 0.64 ± 0.089
333	0 // 2.9	28 ± 4.3 // 22 ± 0.91	0.71 ± 0.036 // 0.88 ± 0.041
343	0 // 2.9	22 ± 1.0 // 18 ± 1.0	0.66 ± 0.027 // 0.83 ± 0.063
353	0 // 2.9	19 ± 1.0 // 15 ± 0.91	0.65 ± 0.045 // 0.72 ± 0.032

a The values and errors originate from 3–4 consecutive cycles for each experiment.

The maximum capacity at RH 0% was 1.3 mmol·g^–1^ (298 K), and the minimum capacity at RH 0% was 0.65 mmol·g^–1^ (353 K). With increasing adsorption temperatures under dry conditions, the CO_2_ breakthrough curves shifted more toward those of N_2_ (Figure S10), indicating a diminishing CO_2_/N_2_ selectivity. Furthermore, the experiments with humidified gas streams yielded a maximum capacity of 0.88 mmol·g^–1^ (RH 15%; 333 K) and minimum of 0.56 mmol·g^–1^ (RH 90%; 298 K). The ratio of the CO_2_ capacity at *P*(H_2_O) = 2.9 kPa over the CO_2_ capacity at *P*(H_2_O) = 0 kPa is plotted in Figure 2C,D. The horizontal dashed line at 1.0 represents the dividing line below which water negatively affects CO_2_ adsorption and above which water-enhanced CO_2_ adsorption is observed. At 298 K at 90% RH only 42% of the original CO_2_ capacity under dry conditions is retained. It is clear that from a certain relative humidity onward (between RH 39%, *T* = 313 K and RH 15%, *T* = 333 K), water strongly competes with CO_2_. This transition coincides with the onset of water cluster formation at ∼30% RH in the water vapor sorption isotherms. In addition, we observed that RH values of 6, 9, and 15% at adsorption temperatures of 353, 343, and 333 K, respectively, increased the CO_2_ adsorption capacity of TAPB-NDA-COF up to ∼1.3 times. The CO_2_/N_2_ selectivity is increased going from 298 K at 90% RH where the CO_2_ and N_2_ breakthrough curves nearly overlap, to lower RH values/higher temperatures where a clear shift between the curves is again noticeable. Finally, although a detailed study concerning the hydrolytic stability of COFs as adsorbents is not the main focus of this research, we duplicated the breakthrough experiment at 298 K and RH 0% after all breakthrough experiments (involving a total exposure time of >75 h and temperatures up to 403 K) presented here had been executed. We overlapped these curves in Figure S13 and noticed no considerable change in CO_2_ separation performance.

**Figure 3:**
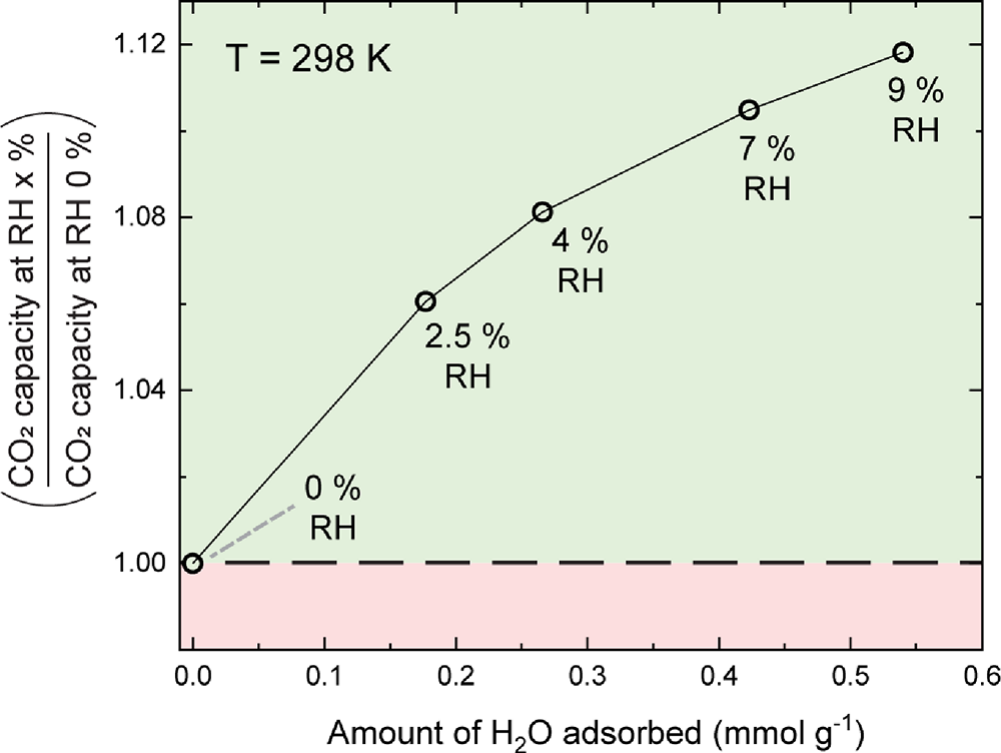
Ratio of the CO_2_ capacity at specific RH values over the CO_2_ capacity at RH 0%, plotted as a function of the amount of H_2_O adsorbed, at 298 K and atmospheric pressure. Capacity values are extracted from CO_2_ and H_2_O sorption TGA experiments based on a protocol proposed by Llewellyn and co-workers.^9^ The shown % RH are those that correspond in equilibrium with the amount of adsorbed water as determined from the water adsorption isotherm at 298 K.

To also understand the CO_2_ adsorption at constant temperature and pressure (298 K, atmospheric pressure) for varying amounts of adsorbed water, we opted for a small-scale laboratory test based on a protocol developed by Llewellyn and co-workers,^9^ where a pre-humidified COF sample was subjected to CO_2_ adsorption and desorption cycles in a thermogravimetric analysis (TGA) instrument. Between the cycles, small amounts of water were successively desorbed, in order to monitor the CO_2_ capacity of the material at various water loadings (see the Supporting Information and Figure S14 for the complete protocol and data analysis). The results are summarized in Figure 3, where the data was normalized over the specific CO_2_ capacity in dry conditions. The relative humidity corresponding in equilibrium with the different amounts of adsorbed water (determined from the water adsorption isotherm at *T* = 298 K) is also indicated. The measurable range of adsorbed water content with this method is restricted, since the weakly adsorbed water is desorbed quickly by the dry passive nitrogen flow. We see the CO_2_ capacity increasing with the adsorbed water content, up to the highest measurable content, which corresponds with ∼9% RH. Likely the relative humidity corresponding to the maximum amount of CO_2_ adsorbed is even higher. These data indicate a synergistic effect of pre-adsorbed water enhancing the CO_2_ capacity of the COF compared to the ‘dry’ state at a constant temperature, strengthening the notion that the key parameter for enhanced CO_2_ uptake is relative humidity. Similar effects have been noticed by Ibarra and co-workers,^14,15^ among others, and a comparison between the CO_2_ capture performance (dry and humid) of TAPB-NDA-COF and other adsorbents under similar experimental conditions is provided in Table S2. Here, mainly MOFs with μ-OH segments in their structure were compared as these MOFs show enhanced CO_2_ uptake at comparable low amounts of pre-adsorbed water. The performance of another MOF, HKUST-1 bearing open metal sites that are occupied by water at low humidity levels, is also compared. The common structural feature that causes enhanced CO_2_ adsorption seems to be hydrophilic groups (be it μ-OH or open metal sites), which – once bound to water molecules – favor CO_2_ binding more than the dry structures themselves.

**Figure 4:**
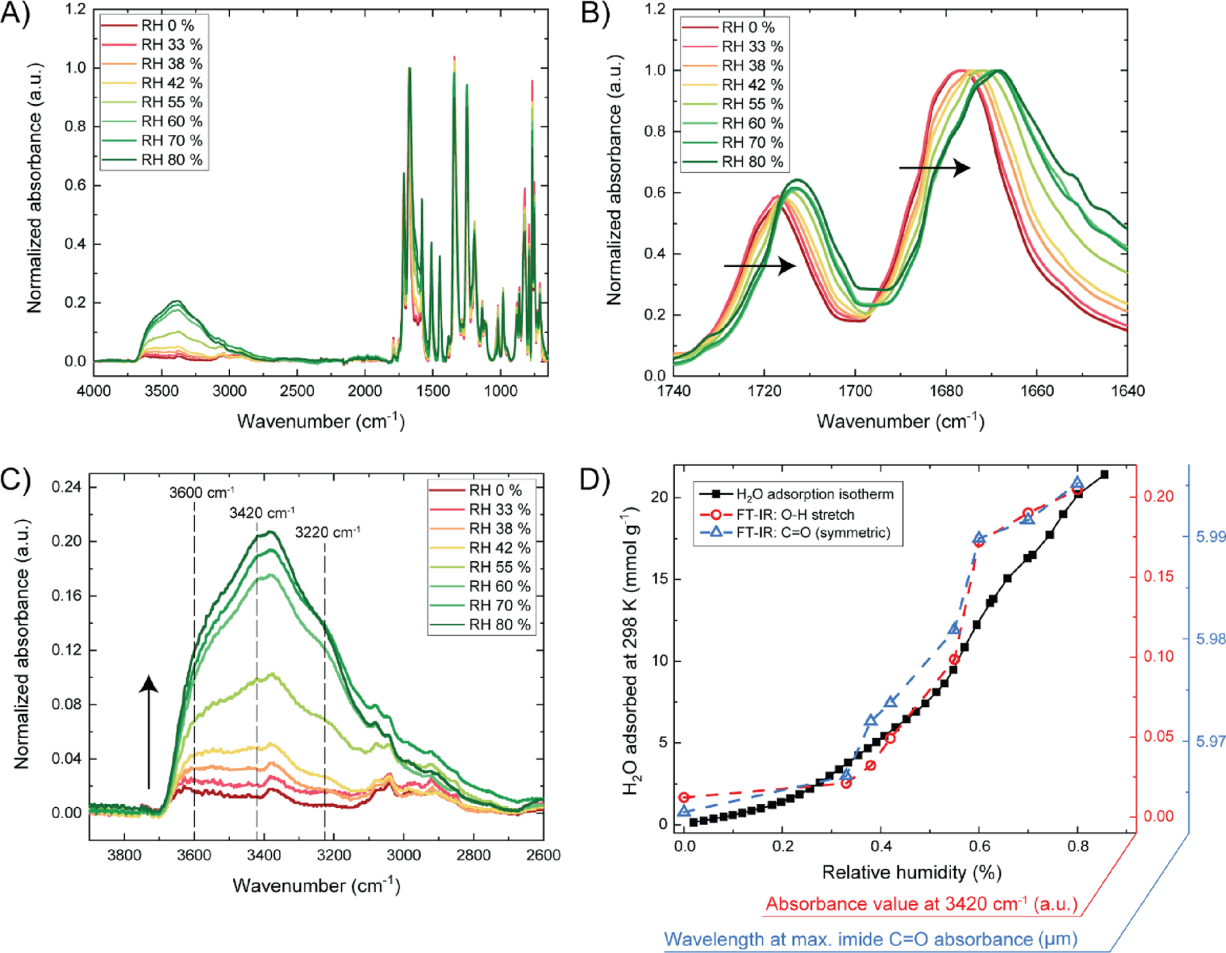
(A) FT-IR spectra of TAPB-NDA-COF equilibrated at 298 K and various RH values. (B) Zoom in on the (a)symmetric C=O stretch vibrations of the imide bond. (C) Zoom in on the region of O–H stretch vibrations. (D) Overlap of water vapor sorption isotherm at 298 K, a plot of the inverse of the imide carbonyl peak maxima (1677–1668 cm^–1^, extracted from panel (B)) as a function of the RH and a plot of RH versus the absorbance values at 3420 cm^–1^, extracted from panel (C).

To relate these findings to the structure and chemistry of the TAPB-NDA-COF, we performed *ex situ* FT-IR experiments at controlled RH values. Figure 4A represents the FT-IR spectra for various COF aliquots equilibrated at 298 K and specific RH values. The spectra were normalized over the symmetric imide carbonyl stretching vibrations at maximum absorbance in the region of 1640–1700 cm^–1^. We varied the RH from ∼0% to 80% and performed a control experiment where the COF exposed to RH 80% was vacuum degassed again (to RH ∼ 0%) and subjected to FT-IR measurement. The spectra of prior to and after water equilibration overlapped (Figure S15), indicating that the changes observed here are reversible. It should be noted, however, that the samples denoted as RH 0% are briefly exposed to the humidity of the lab during transfer to the ATR crystal and the FT-IR spectrometer.

The imide peaks that we investigated are presented in Figure 4B since these signals changed (red-shifted) most drastically as a result of increasing RH values, which suggests that these groups are actively involved in the water uptake. This shift (even at low water loadings: 3 cm^–1^ from 0 to 38% RH) is significant, considering that within a COF unit cell, there is a large amount of active sites: ∼ 8.6 mmol carbonyl O atoms per gram of COF. Both the symmetric (∼1680 cm^–1^) and asymmetric (∼1720 cm^–1^) carbonyl stretching vibrations of the imide bonds are red-shifted as a result of increasing RH values. Focusing on the symmetric vibrational mode, the maximum absorbance shifts significantly from 1677 cm^–1^ at RH 0% to 1668 cm^–1^ at RH 80%. Although the effect was less pronounced on the C–N stretch vibration of the imide bond (Figure S16), the increasing RH values caused a noticeable blue-shift from 1340 cm^–1^ at RH 0% to 1344 cm^–1^ at RH 80%. The lone pair of the imide nitrogen is delocalized through resonance with the imide carbonyls, making the nitrogen less likely to participate in hydrogen bonding, in line with the observation here that the C–N bond strength increases upon water adsorption. Second, the absorbance intensity of the O–H stretching vibrations increases significantly with larger RH values (Figure 4C). While this region is a complex accumulation of different vibrational modes, we focused on three main signals: ∼ 3600, 3420, and 3220 cm^–1^. All three signals are RH-dependent, but the onset of their increase seems to differ. The signal at ∼3600 cm^–1^ is already present at ∼0% RH and seems to be dominating at low RH values. The signals at 3420 and 3220 cm^–1^ develop into a clear peak and shoulder peak, respectively, at higher RH values (around 38–42%). Overall the ratio of the peak at ∼3600 cm^–1^ compared to the one at 3220 cm^–1^ is much higher at RH up to 42%, versus at higher values. In Figure 4D, we plotted both the increasing absorbance of the O–H stretching vibration at 3420 cm^–1^ and the inverse of the imide carbonyl peak maxima (1677–1668 cm^–1^) as functions of the RH. In the same plot, we display the water isotherm of the COF at 298 K (also following a water signal over a RH range, albeit manometrically) as complementary data to the FT-IR data.

Solid-state NMR studies on hydrated polyimide films by Waters *et al.* support the claim of imide-carbonyl groups being the main active sites for water adsorption.^21^ Moreover, the detailed work of Musto *et al.* on the interaction of water with polyimides studied by 2D-FT-IR correlation spectra noticed the imide carbonyl red-shift as well^22^ and distinguished first- and second-shell hydration layers through deconvolution of the complex O–H stretch region. The first hydration event generates single site adsorbed water of which the free O–H bond vibrates at a relatively high wavenumbers. Indeed, the shoulder peak at 3600 cm^–1^ is more prominent at low RH values, compared to the signals at 3420 and 3220 cm^–1^ (Figure 4C). These latter two signals could represent multiple-bound water in the form of water clusters and such clusters seem to become more significant around 38–42% RH. Furthermore, the imide-carbonyl signals red-shift continuously over the whole RH range (following a similar trend to the water vapor isotherm; Figure 4D). Thus, while they are the main adsorption sites for water (∼ 8.6 mmol per gram of COF), they are not completely saturated upon the first hydration events. This stands in contrast to, for example, MOFs with hydrophilic open metal sites. There, a clear 2-step mechanism can be observed: (1) coordination of water on active sites and subsequent saturation of these sites and (2) pore filling through water network formation.^23^ In the case of TAPB-NDA-COF, however, it seems more likely that at 38–42% RH, water–water interactions are roughly equally favored as water-framework interactions, *i.e.*, isolated water-carbonyl interactions in one pore occur simultaneously with water clustering and center pore filling in the other pore.^24^ Such a pore filling mechanism allows rationalization of the breakthrough results. The RH values where single-site adsorbed water is a dominant feature namely coincide with the RH values where water-enhanced CO_2_ adsorption was observed. Therefore, it is likely that the Δ*H*_ads_ for CO_2_ on single-site adsorbed water is greater than the Δ*H*_ads_ for CO_2_ on the adsorption sites of the dry COF. Recent computational studies using many-body potential energy functions for simple (H_2_O)_*m*_(CO_2_)_*n*_ systems revealed that the interaction energies of clusters with *m* ≥ 1 are always greater than for clusters where *m* = 0 (*e.g.*, (CO_2_)_2_ ∼ −6.3 kJ·mol^–1^ while (H_2_O)(CO_2_) ∼ −12.4 kJ·mol^–1^).^25^ These absolute values cannot be fully translated to our system (partly due to the inclusion of a complex adsorbent), yet the relative trends of our study and these computational results coincide and strengthen the hypothesis of water-enhanced CO_2_ adsorption being attributed to single-site adsorbed water. The interaction between this single-site adsorbed water and CO_2_ can occur via two modes: between the partially negatively charged oxygen of H_2_O and the partially positively charged carbon of CO_2_ or between the partially positively charged hydrogen of H_2_O and the partially negatively charged oxygen of CO_2_.^26,27^ This particular interaction is likely to be energetically more favorable than the interaction between the dry COF and CO_2_. As such, we expect that CO_2_ uptake could be enhanced by pre-confining other molecules as well,^28^ especially those with similar hydrogen bonding capabilities.

Lastly, the rate of the CO_2_–COF mass transfer is slower in the breakthrough experiments that showed water-assisted CO_2_ adsorption, *i.e.*, slower diffusion through the porous network. The slower CO_2_ diffusion in sub-nanoporous channels created at similar water concentrations has also been observed in MOF UiO-66.^11^ As also mentioned in their research, water-enhanced effects at equilibrium can come at the cost of slow kinetics, which shows the importance of fundamentally understanding these processes for rapid separation processes.

## 3 Conclusions

In summary, the extensive breakthrough studies allowed us to couple material characteristics to the COF’s performance of CO_2_ separation from humid CO_2_/N_2_ streams. The relative humidity throughout these experiments was varied at constant water vapor pressure of around 2.9 kPa in the gas stream by changing the adsorbent column temperature. By doing so, we discovered that the CO_2_ adsorption capacity increases by ∼25% in comparison with the dry stream at 333 K (RH = 15%) and 343 K (RH = 9.3%), when – in the case of TAPB-NDA-COF – the gas stream is humidified. Via measuring the CO_2_ adsorption capacity for varying amounts of adsorbed water at a constant temperature (298 K) using a thermogravimetric protocol, we corroborated the water-enhanced CO_2_ adsorption effect. These results show that this phenomenon for the TAPB-NDA-COF is robust (independent of the experimental setup).

Via infrared spectroscopy, we could correlate the enhanced CO_2_ adsorption at low relative humidity values with single-site adsorbed water on the imide carbonyl groups. The Δ*H*_ads_ for CO_2_ adsorbed on such a water adsorbate is likely greater (more negative) than the Δ*H*_ads_ determined for the CO_2_ interaction with dry the COF (∼ −35 kJ·mol^–1^), resulting in water-enhanced CO_2_ adsorption. At 38–42% RH, water clustering and center pore filling becomes dominant. Here, CO_2_ binding would mean the interruption of multiple hydrogen bonds (a large energy penalty), and thus CO_2_ adsorption is inhibited. Percolation of the water network at these higher RH values drastically lowers the CO_2_ capacity of the COF.

Although there are many examples displaying the water tolerance of COFs, few place this in the context of how adsorbed water affects CO_2_ capture and separation,^29,30^ and fewer still systematically varied RH values in industrially relevant breakthrough setups (where mostly a negative effect of water is observed).^6,7^ The results presented here are among the first to show cooperative CO_2_–H_2_O adsorption in COFs. The overall CO_2_ adsorption capacity of this COF is rather modest (∼1.3 mmol·g^–1^ at 0.6 bar CO_2_). Yet, we trust that the understanding of water-enhanced CO_2_ adsorption we uncovered here can be exploited via the tunable nature of COFs, specifically focusing on COFs that show a high CO_2_ adsorption capacity.

## Supporting Information Available

Reagents, characterization techniques, synthesis of TAPB-NDA-COF, isosteric enthalpy of adsorption calculation, water-COF FT-IR binding studies protocol, breakthrough studies experimental and analysis, TGA protocol, FT-IR data (monomers, COF, extra data for water studies), standard TGA curve COF, PXRD COF, fitting curve for PSD, isosteric enthalpy of adsorption curve and fitting parameters, supplementary breakthrough curves, supplementary TGA data (PDF)
