# Supplementary material for: Competitive and Cooperative CO2–H2O Adsorption through Humidity Control in a Polyimide Covalent Organic Framework
Source: ACS Appl Mater Interfaces. 2023 Jun 9;15(24):29186–94. doi: 10.1021/acsami.3c04561 (PMC10288428; doi:10.1021/acsami.3c04561)
Supplement: Supplementary file 1 — am3c04561_si_001.pdf [file am3c04561_si_001.pdf]

Supporting information

Competitive and Cooperative CO_2–H_2O Adsorption Through Humidity Control in a Polyimide Covalent Organic Framework

Hugo Veldhuizen, Saira Alam Butt, Annemiek van Leuken, Bart van der Linden, Willy Rook, Sybrand van der Zwaag, Monique A. van der Veen*

*corresponding author: M.A.vanderVeen@tudelft.nl

Department of Novel Aerospace Materials, Delft University of Technology, 2629 HS Delft, The Netherlands

Department of Catalysis Engineering, Delft University of Technology, 2629 HZ Delft, The Netherlands

Table of contents

Experimental method details S-3

Reagents S-3

Characterization techniques S-3

Synthesis and processing of TAPB-NDA-COF S-3

Isosteric enthalpy of adsorption S-4

Water-COF FT-IR binding studies S-4

Breakthrough studies S-4

TGA measurements of COFs with pre-adsorbed water S-7

Supplementary experimental data S-8

FT-IR: monomers and COF S-8

TGA S-8

PXRD S-9

BETSI S-10

PSD fitting curve S-11

Cumulative pore volume S-11

Isosteric enthalpy of adsorption S-12

Supplementary breakthrough data S-13

Supplementary TGA data on COFs with pre-adsorbed water S-17

Supplementary FT-IR data on water-COF binding studies S-19

Experimental method details

Reagents

All reagents presented in this report were commercially available and used without further purification or treatment. 1,3,5-Tris(4-aminophenyl)benzene (≥ 93 %) and isoquinoline were purchased from TCI Europe N.V (Zwijndrecht, Belgium), 1,4,5,8-naphthalenetetracarboxylic dianhydride (≥ 95 %) and ortho-dichlorobenzene from abcr GmbH (Karlsruhe, Germany) and N-methyl-2-pyrrolidone from Acros Organics B.V.B.A (Geel, Belgium).

Characterization techniques

FT-IR spectra were recorded on a PerkinElmer Spectrum 100 FT-IR Spectrometer with an universal ATR accessory over a range of 4000 to 650 cm^{-1}. TGA analyses were performed from 30 to 860 °C, under a nitrogen atmosphere at a heating rate of 10 $°C·min^{-1}$ using a Perkin Elmer TGA 4000. Before the measurement, the samples were degassed at 130 °C for one hour under a nitrogen atmosphere. PXRD patterns were measured on a Rigaku MiniFlex 600 powder diffractometer using a Cu-Kα source (λ = 1.5418 Å) over the 2θ range of 2 ° to 40 ° with a scan rate of 1 $°·minute^{-1}$. Nitrogen and carbon dioxide isotherms were measured on the Micromeritics TriStar II porosity analyser. Water vapour isotherms were measured on the Micromeritics 3Flex adsorption analyser. Prior to the sorption measurements, all samples were degassed at 130 °C under vacuum for 16 h. The Quantachrome VersaWin software package was used for calculations of pore size distributions by fitting the nitrogen adsorption isotherms to the quenched solid density functional theory (QSDFT) carbon model (using slit/cylindrical/spherical pores). No smoothing factor was applied for the PSD calculation.

Synthesis of TAPB-NDA COF

TAPB (351 mg, 1.0 mmol) and NDA (402 mg, 1.5 mmol) were added to a glass cylindrical reactor and subsequently o-DCB (10 mL), NMP (10 mL) and isoquinoline (0.1 mL) were added. Thereafter, the reactor was sealed, vacuum degassed, and placed in an oil bath. Under gently shaking the reactor vessel, it was heated to a temperature of 150 °C. Upon complete dissolution of the monomers, the temperature was step-wise (10 °C per 15 min) increased to 190 °C and kept without agitation at that temperature for 3 days. The reaction mixture was cooled to room temperature, suspended in 60 mL methanol, and mixed thoroughly. The solid was separated from the liquid through centrifugation (10 min at 4400 rpm), after which the solid was washed with methanol (3 x 30 mL) and acetone (1 x 30 mL). After drying the solid in a vacuum oven at 60 °C for one hour, it was subjected to Soxhlet extraction with THF for 20 hours. After that, the COF

was allowed to dry in a vacuum oven at 60 °C for 20 hours. The TAPB-NDA-COF was isolated as an ochre-brown, fluffy powder (610 mg, 87 %).

Post-synthetic processing

The COF powder was pelletized in batches of ~ 100 mg, using a pellet die with a diameter of 20 mm. For each batch, a hydraulic press was used to apply a pressure of 31 MPa for 60 seconds. After that, the pellets were crushed and then, through sieving, we collected COF pellets in the particle size range between 300 and 425 μm. These pellets were used without further treatment for characterization and breakthrough studies.

Isosteric enthalpy of adsorption,

The common Freundlich–Langmuir fit/Clausius–Clapeyron approach for the calculation of ΔH_{ads} was executed according to the procedure described by Alexander Nuhnen and Christoph Janiak (DOI: 10.1039/D0DT01784A) and applied to all CO_2 adsorption isotherms. The corresponding Freundlich–Langmuir fitting parameters are presented in Table S1.

Water-COF FT-IR binding studies

Around 5 mg of pelletized COF was transferred to an open vial. This vial was placed in a 250 mL glass jar that also contained a vial of ~ 10 mL of salt solutions / suspensions. A relative humidity sensor was added to the system, which was subsequently sealed with Parafilm. The COFs were allowed to equilibrate for 16 hours at room temperature. Thereafter, they were immediately subjected to standard FT-IR analysis. The following RH values were monitored for the specific salt solutions: calcium chloride for 33 %, magnesium chloride for 38 %, sodium iodide for 42 %, sodium bromide for 55 %, magnesium bromide for 60 %, sodium chloride for 70 % and distilled water for 80 % relative humidity. The latter value is relatively low compared to other systems with distilled water, but this is likely a kinetic effect (*i.e.* insufficient equilibration time). On the other hand, the lower RH values agree with literature values. RH values of ~ 0 % are obtained by directly retrieving the COF vial from a vacuum oven set at 130 °C. Those vials were directly sealed when retrieved from the oven and utilized directly for FT-IR analysis.

Breakthrough studies

Setup

The breakthrough setup for multicomponent breakthrough studies is an in-house built setup (see Fig. S7), extensively used in the doctoral thesis *Gas Adsorptive Separation through Microporous Materials*, by Andres Garcia, Eduardo, 2019 (https://doi.org/10.4233/uuid:cfbabbfc-b66c-4279-b717-df9e73ed921f). It

primarily consists of He, N_2 and CO_2 feed flows (regulated by mass flow controllers, Brooks 5850 series), water saturators and a packed bed column within which temperature and pressure can be regulated. The bed height of the COF (95 mg) in the stainless steel column (length 79 mm, inner diameter 4 mm) is 39 mm, and the bed is sandwiched between two layers of quartz wool. A four-way switching valve allows the operator to switch between N_2 (at 8 mL·min^{-1}) / CO_2 (at 2 mL·min^{-1}) mixture (feed 1) and He (feed 2) gas streams and back-pressure regulators ensure a constant pressure when switching. The second He flow (at 10 mL·min^{-1}) is added after the column to prevent flow disruptions and securing a constant flow to the analysis instruments. The breakthrough response is monitored by a mass spectrometer (MS, LPM T100 Gas Analyzer) and a relative humidity sensor (PosiTector Dew Point Meter, DeFelsko). A complete overview of the setup is displayed in Figure S7.

Experiments

Prior to every experiment (set of 4 cycles per experimental parameter set), the system is flushed overnight (~ 16 h) with 10 mL·min^{-1} He and the oven is set to 130 °C. Then, the column is allowed to reach the specific temperature necessary for the experiment. An additional pre-treatment step is used for humid experiments, where the column is exposed to a humid He stream with a controlled RH value and temperature, and equilibrated overnight (~ 16 h). Typically, 60 minutes are used for the adsorption and desorption cycles unless stated otherwise. Dead volume measurements were performed by replacing the COF with non-adsorbent silicon carbide (350 µm) using a similar bed height.

Analysis of breakthrough data

MS signal to flow rate

The conversion of MS signal to estimated flow rates from the exit are based on the following equations. For every component x, a normalized component fraction y is calculated (eq. 1) based on the MS signal, noise and final steady state (ss) values (i.e. when breakthrough of all components has occurred and the signals stabilized). The *noise* signal is the background MS signal.

$$y_x(t) = \frac{signal_x(t) - noise_x}{signal_x(ss) - noise_x} \qquad \text{eq. 1}$$

At steady state, the calculated exit flow rates are directly proportional to $y_x(t)$:

$$F_x(ss) = y_x(t) \cdot F_x^{feed} \qquad \text{eq. 2}$$

With F_x^{feed} being the feed flow rates of components x.

But, to have an expression for $F_x(t)$ we have to use a correction factor: there is a constant flow going into the MS, but the total flow rate that exits the adsorption column changes over time (due to adsorption / desorption of different components). So, the time-dependent correction factor $C(t)$ should correct for the fact that the total flow rate at steady state can be different than the total flow rate during adsorption:

$$F_x(t) = y_x(t) \cdot F_x^{feed} \cdot C(t) \qquad \text{eq. 3}$$

$$F_x(t) = y_x(t) \cdot F_x^{feed} \cdot \frac{F_{N2}(t) + F_{CO2}(t) + F_{He}(t)}{F_{total}(ss)} \qquad \text{eq. 4}$$

While eq. 4 can be written as three separate equations for all components, these three equations are not independent and thus cannot be solved in order to get an expression for $F_x(t)$ that is defined by only known values. We also cannot assume $F_{He}(t)$ to be constant (which would help solving the equations), as it is used both as a flush and a sweep gas. Instead, we found that the equation 5, using only known values, where the term $F_{N2}(t) + F_{CO2}(t) + F_{He}(t)$ is approximated as $\left(y_{N2}(t) \cdot F_{N2}^{feed}\right) + \left(y_{CO2}(t) \cdot F_{CO2}^{feed}\right) + \left(y_{He}(t) \cdot F_{He}^{feed}\right)$ yields corrected breakthrough curves. In this case, features such as the roll-up effect on the nitrogen curves are visible and calculating capacities based on eq. 5 yields comparable capacity values to the ones based on the TGA experiments.

$$F_x(t) = y_x(t) \cdot F_x^{feed} \cdot \frac{\left(y_{N2}(t) \cdot F_{N2}^{feed}\right) + \left(y_{CO2}(t) \cdot F_{CO2}^{feed}\right) + \left(y_{He}(t) \cdot F_{He}^{feed}\right)}{F_{total}(ss)} \qquad \text{eq. 5}$$

The normalized corrected flow for component x is defined as $\frac{F_x(t)}{F_x^{feed}}$, otherwise written as $\left(\frac{\mathrm{F}(x)}{F_0(x)}\right)$ in the main manuscript.

CO$_2$ breakthrough time and capacity

The CO_2 breakthrough time is classified as the breakthrough time difference between N_2 and CO_2 breakthrough. In this research, we identify the exact point of breakthrough when there is a significant difference in the slope of the curve (see Figure S11). CO_2 capacities were calculated from their breakthrough curves (time vs F(CO_2)), by integrating the area above this curve. Areas were always integrated using:

- the x-axis range: time t = 0 (defined as N_2 breakthrough) to t = 1200 seconds (chosen as here typically all curves have plateaued, see Figure S11), and
- the y-axis range: F(CO_2) = 0 to F(CO_2) = 2.

Then, this area is subtracted by the integrated area above the CO_2 breakthrough curve from the silicon carbide experiments (dead volume). The dry-silicon carbide area was used for the dry COF experiments,

and the humid-silicon carbide area for the humid COF experiments. The value of the final integrated area is divided by the weight of the COF in the column (95 mg) to obtain the CO_2 capacity in $cm^3 \cdot g^{-1}$ (at STP) or $mmol \cdot g^{-1}$ (Table 1).

TGA measurements of COFs with pre-adsorbed water

The protocol for TGA measurements of COFs with pre-adsorbed water was adopted from Llewellyn and co-workers with minor changes (https://doi.org/10.1002/cssc.201601816). The pressure during these experiment is kept constant at atmospheric values. First, COFs were equilibrated in a closed vessel in the presence of water at 80 % RH. Then, ~ 7 - 10 mg of this powder was transferred to the TGA crucible and submitted to the following program:

1. 100 $mL \cdot min^{-1}$ N_2 flow, 298 K, 60 minutes (equilibration, desorption of weakly bound water)
2. 100 $mL \cdot min^{-1}$ CO_2 flow, 298 K, 30 minutes (adsorption)
3. 100 $mL \cdot min^{-1}$ N_2 flow, 298 K, 30 minutes (desorption)
4. 100 $mL \cdot min^{-1}$ N_2 flow, heating to desired temperature at 10 $K \cdot min^{-1}$ (controlled water desorption)
5. 100 $mL \cdot min^{-1}$ N_2 flow, cooling to 298 K at 10 $K \cdot min^{-1}$
6. 100 $mL \cdot min^{-1}$ N_2 flow, 298 K, 15 minutes (ensure temperature stabilization)
7. 100 $mL \cdot min^{-1}$ CO_2 flow, 298 K, 30 minutes (adsorption)
8. 100 $mL \cdot min^{-1}$ N_2 flow, 298 K, 30 minutes (desorption)

We used water desorption temperatures of 333, 363, 393 and 423 K at step 4. All weights from the TGA measurements were corrected with the weight of the empty crucible following the same protocol. After the four measurement cycles of step 1.-8., the weight of the COF with 0 $mmol \cdot g^{-1}$ H_2O adsorbed was obtained by first drying the COF powder under 100 $mL \cdot min^{-1}$ N_2 flow at 423 K for three hours, cooling to 298 K and stabilizing at that temperature for 60 minutes, after which the monitored weight was taken as the dry-COF weight. Then, the dry COF was again subjected to a final CO_2 ad- and desorption cycle (replicate step 7 and 8). The supplementary data to the main figure (Figure 3) are presented in supplementary Figure S13. The data were analysed as is listed in the supplementary protocol of Llewellyn and co-workers. Similar to the issues with their data analysis, we calculated 2 CO_2 and 2 H_2O capacities (including and excluding the unaccounted weight increase, step 4 in Figure S13A) which were averaged for the final data.

Supplementary experimental data

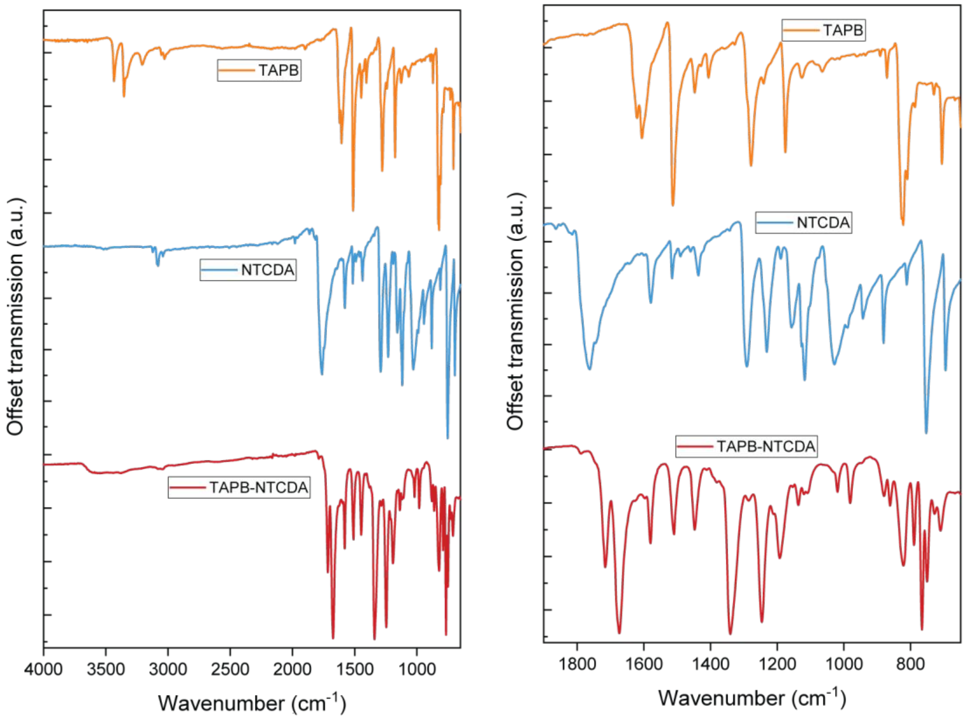

Figure S1. FT-IR spectra of TAPB-NDA-COF and its originating monomers TAPB and NDA. The right graph is a zoom-in of the left graph.

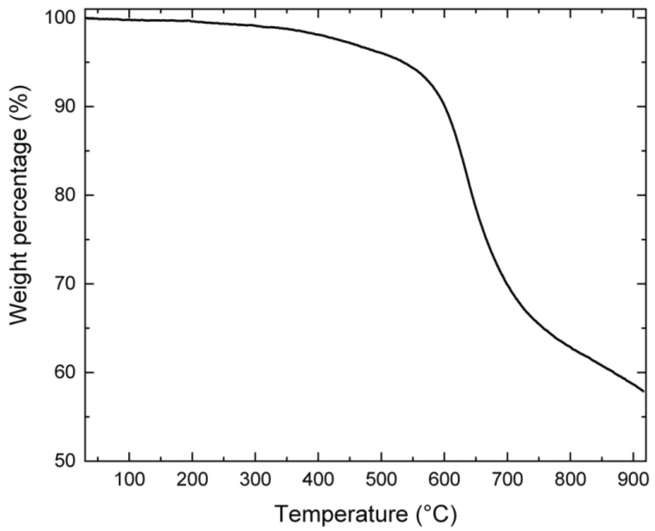

Figure S2. Standard TGA curve of TAPB-NDA-COF. Heating rate of 10 °C/min was used under a constant nitrogen flow at 100 mL/min.

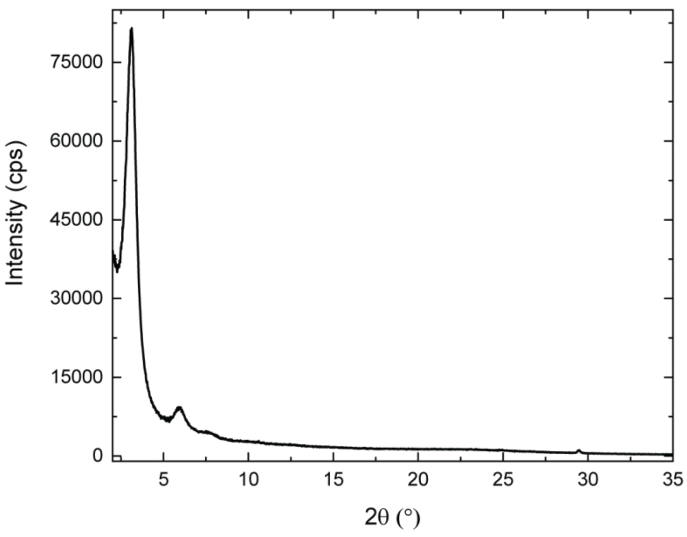

Figure S3. PXRD pattern of TAPB-NDA-COF.

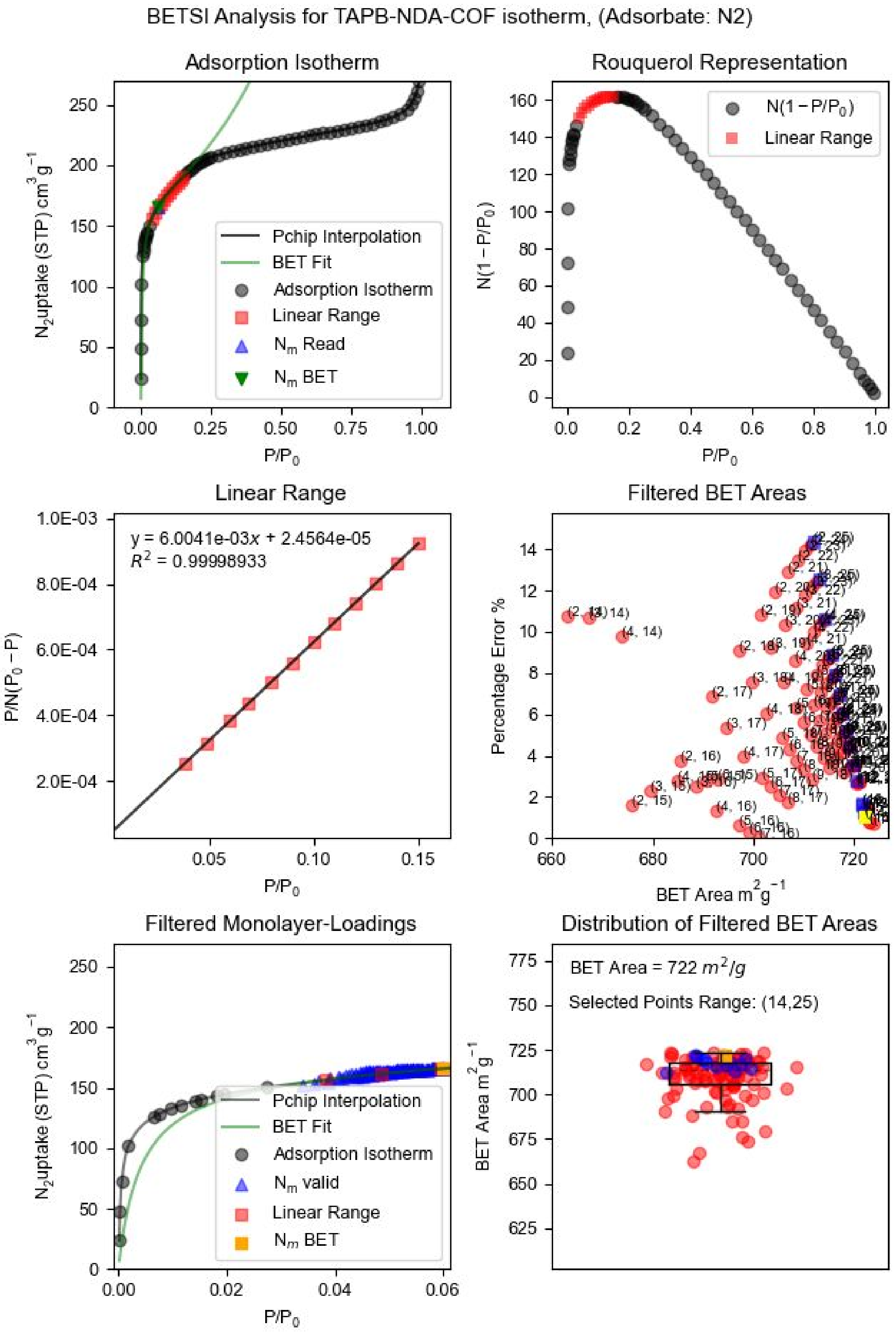

Figure S4. BETSI analysis report for the adsorption isotherm of TAPB-NDA-COF.[S1]

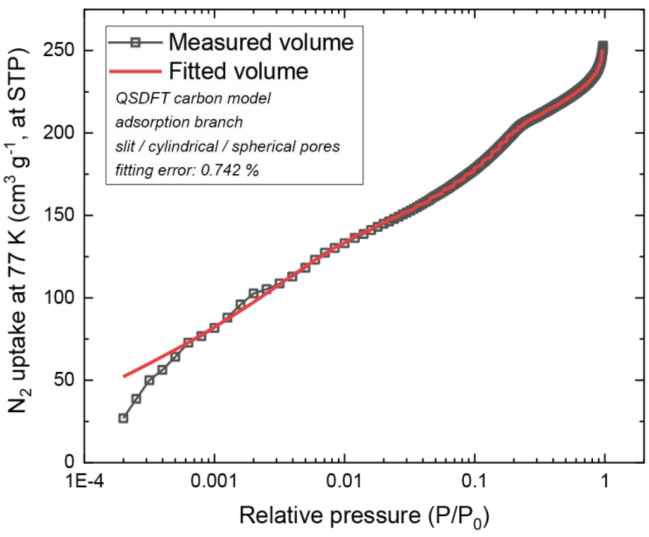

Figure S5. Fitting curve of the PSD of TAPB-NDA-COF, using the adsorption branch of the nitrogen isotherm and a QSDFT carbon model.

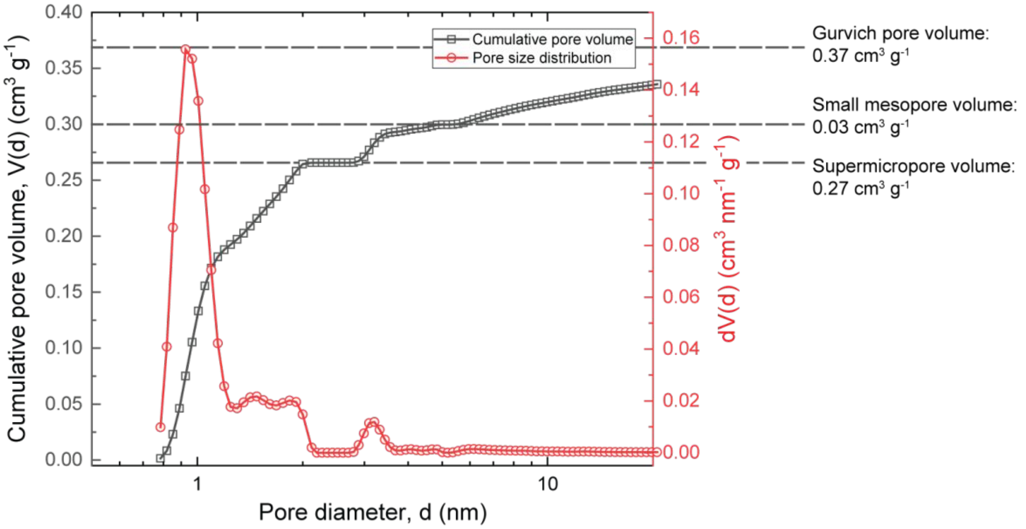

Figure S6. Cumulative pore volume and pore size distribution of TAPB-NDA-COF with indicated supermicropore, small mesopore, and Gurvich pore volume.

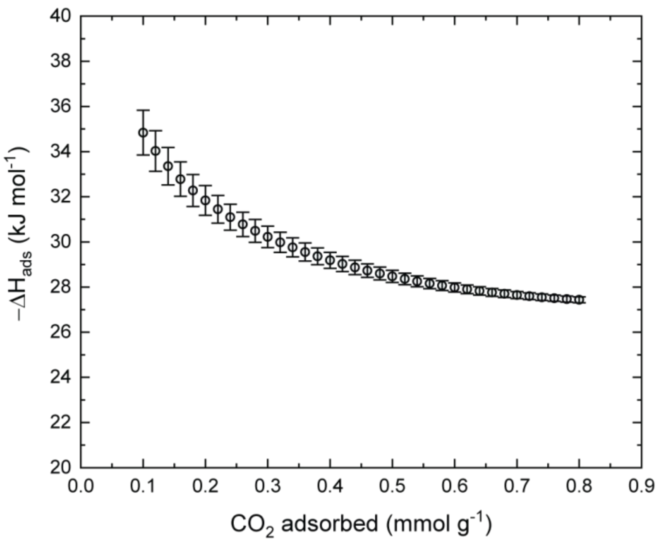

Figure S7. Iosteric enthalpy of adsorption based on the CO_2 adsorption curves and calculated via the Freundlich-Langmuir / Clausius-Clapeyron approach.

Table S1. Fitting parameters for the Freundlich–Langmuir fits applied to CO_2 adsorption isotherms. The equation used for the fit is B = (a*b*p^c)/(1+b*p^c), with p being the absolute pressure (kPa) and B the CO_2 uptake (mmol g^{-1}).

Temperature (K)	a	b	c	Reduced χ^2	R^2
**283**	6.89 ± 0.33	0.01424 ± 4E-4	0.730 ± 0.009	4.80E-5	0.9999
**293**	5.85 ± 0.28	0.0109 ± 3E-4	0.779 ± 0.009	3.15E-5	0.99991
**303**	3.92 ± 0.09	0.0102 ± 1.2E-4	0.845 ± 0.006	7.72E-6	0.99996
**313**	3.59 ± 0.08	0.00745 ± 9E-5	0.876 ± 0.005	3.76E-6	0.99998
**333**	2.99 ± 0.08	0.00414 ± 6E-5	0.936 ± 0.005	1.29E-6	0.99998

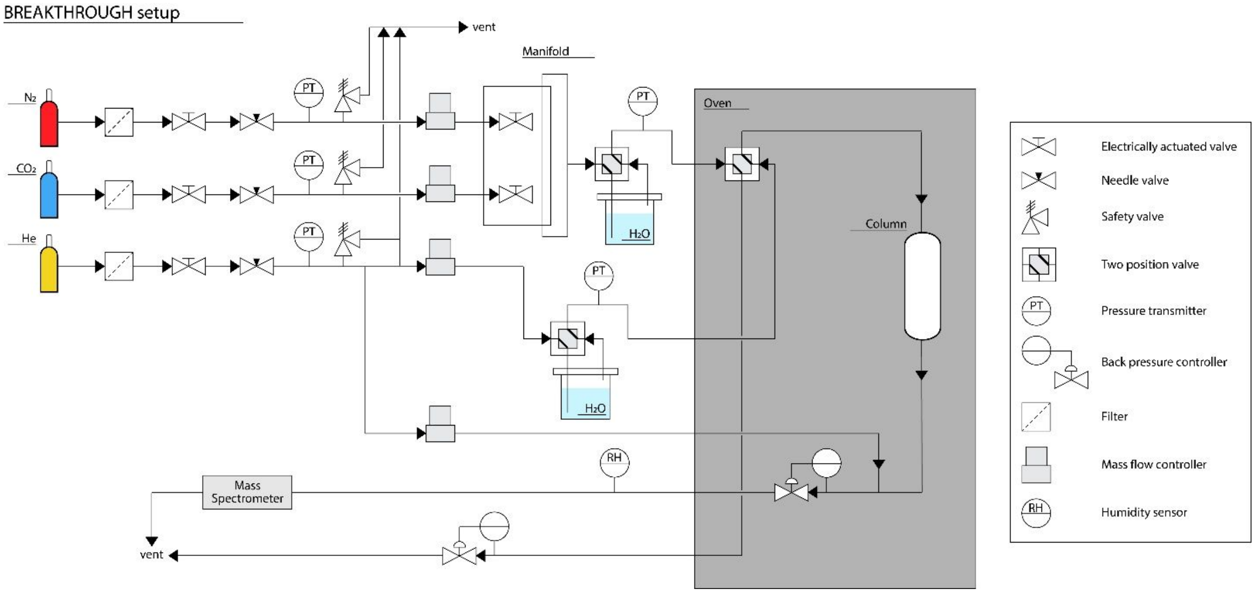

Figure S8. Schematic representation of breakthrough setup.

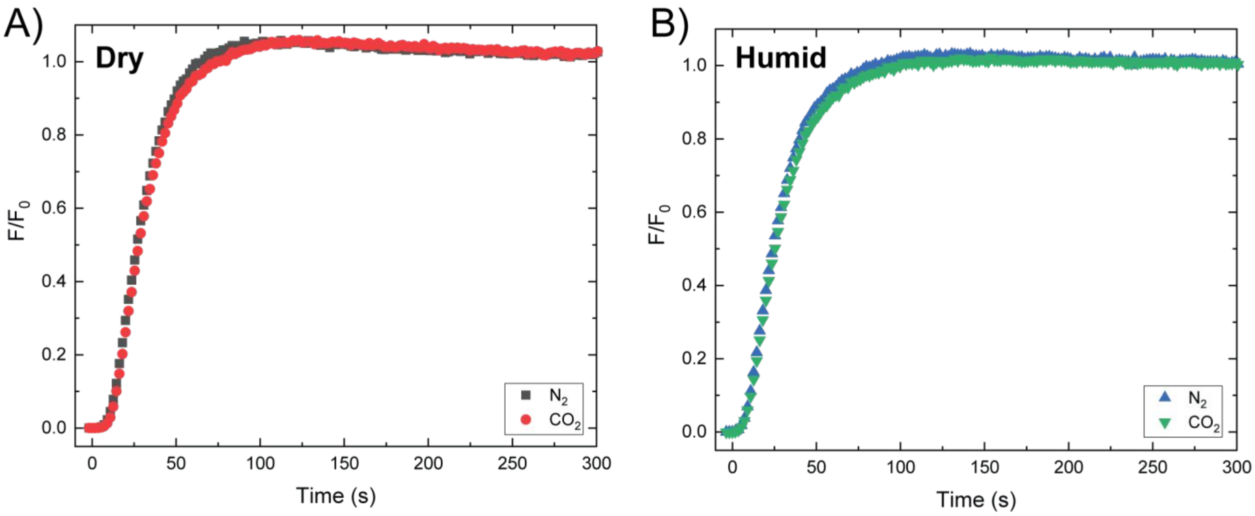

Figure S9. Normalized N_2 (8 mL/min) and CO_2 (2 mL/min) silicon carbide breakthrough curves at 3.1 bar and 298 K under **A)** dry, and **B)** humid conditions.

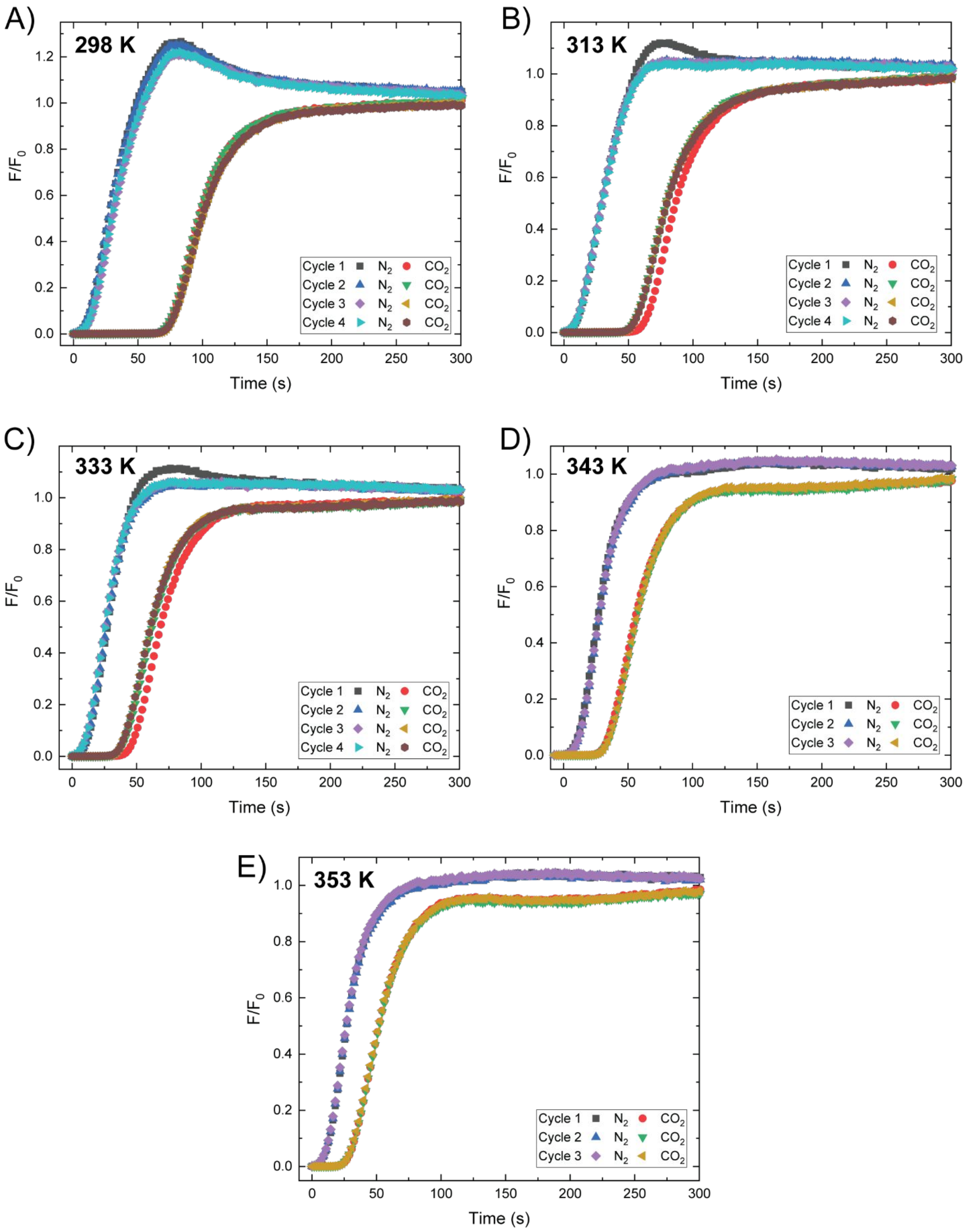

Figure S10. Normalized N_2 (8 mL/min) and CO_2 (2 mL/min) TAPB-NDA-COF breakthrough curves under dry conditions at 3.1 bar and **A)** 298, **B)** 313, **C)** 333, **D)** 343 and **E)** 353 K.

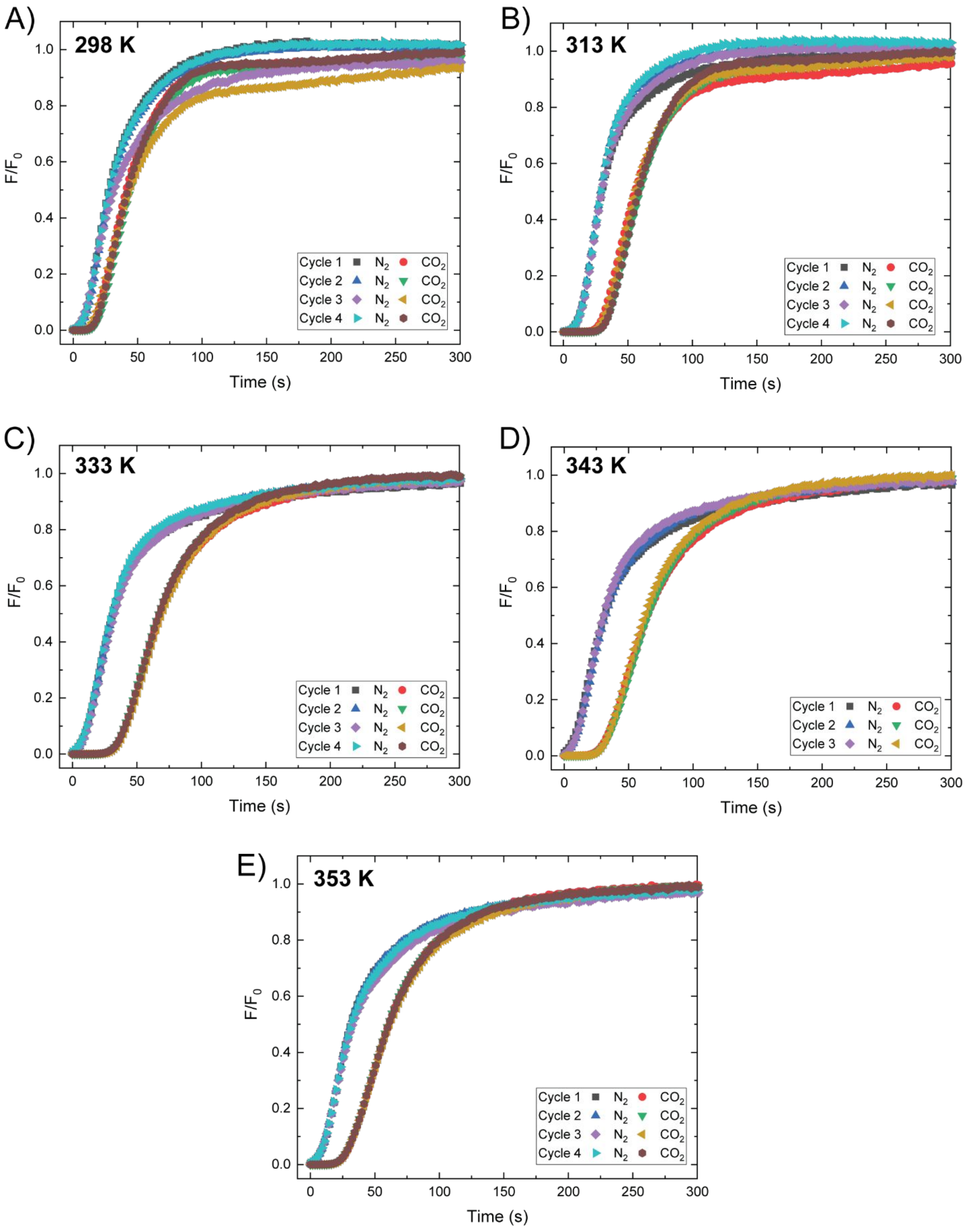

Figure S11. Normalized N_2 (8 mL/min) and CO_2 (2 mL/min) TAPB-NDA-COF breakthrough curves under humid (P_{H2O} = 2.9 kPa) conditions at 3.1 bar and **A)** 298, **B)** 313, **C)** 333, **D)** 343 and **E)** 353 K.

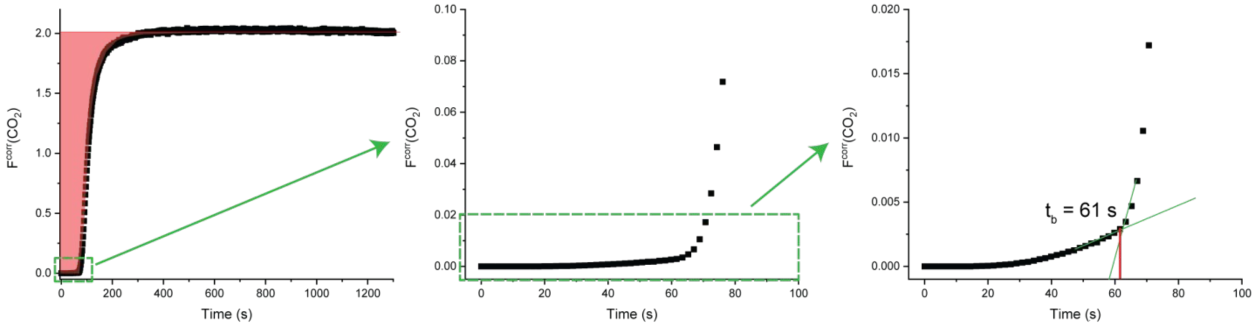

Figure S12. Example CO_2 breakthrough curve (dry experiment, cycle 1) for illustration of how analysis was conducted. Left: light-red area indicates integrated area to determine capacity. Right: the last data point before the slope of the curve starts to significantly increase is taken as the point at breakthrough.

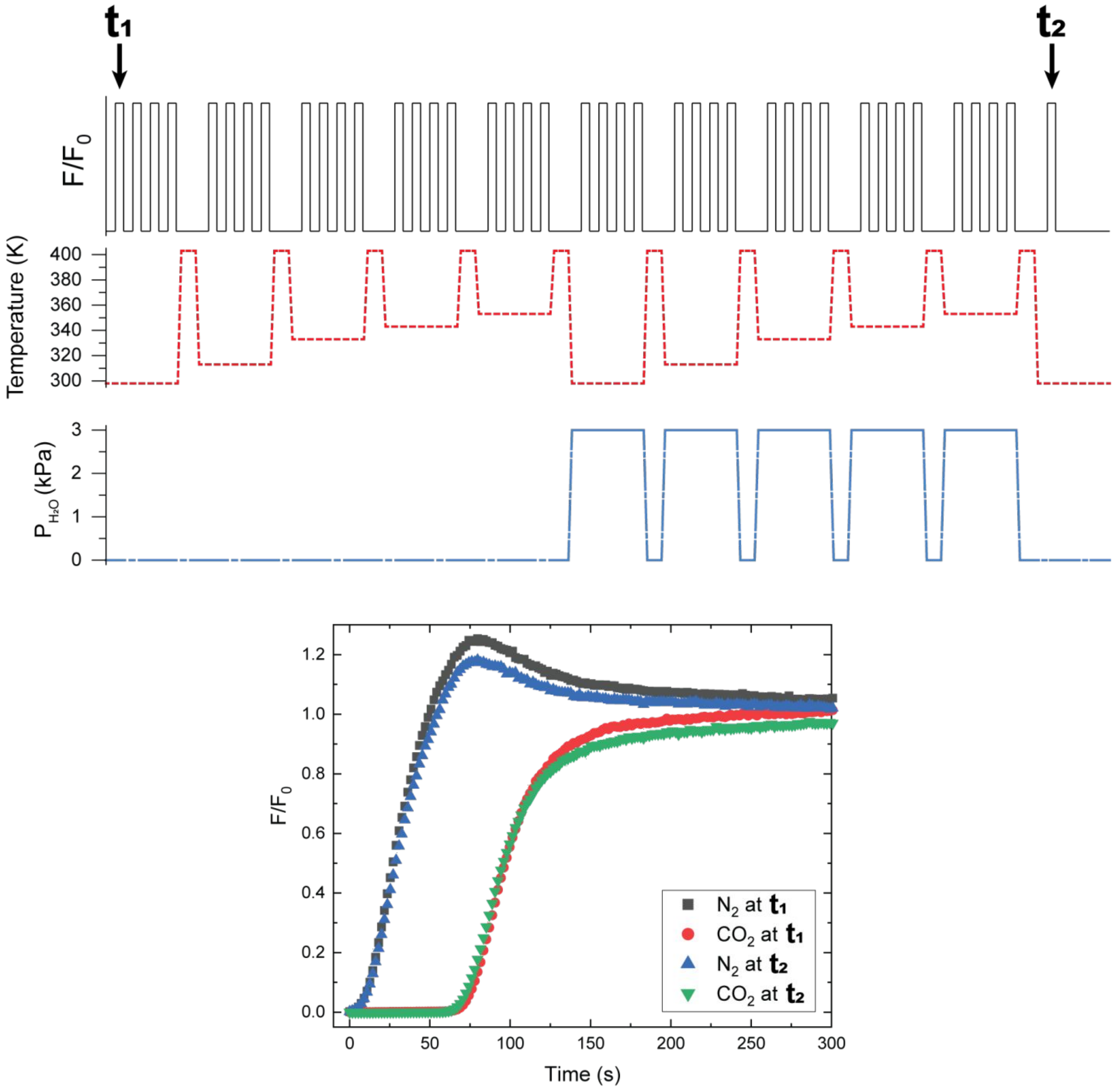

Figure S13. Top: Breakthrough conditions to which a single batch of TAPB-NDA-COF was exposed over time. Bottom: Normalized N_2 (8 mL/min) and CO_2 (2 mL/min) TAPB-NDA-COF breakthrough curves under dry conditions at 3.1 bar and 298 K, comparing t1 and t2 as indicated by the top figure.

TGA measurements of COFs with pre-adsorbed water

Temperatures of 333, 363, 393 and 423 K were chosen to step-wise desorb water and observe how the CO_2 capacity of the material changed as a result of that (Figure S13A). This trend has been visualised in Figure S13B. After all 4 runs had been executed, the COF powder was dried completely by flowing N_2 at 423 K for three hours. Then the powder was cooled to 298 K and stabilized, and its weight was taken as the weight at 0 mmol·g^{-1} H_2O adsorbed. A final CO_2 ad- and desorption cycle was performed to get the CO_2 capacity of the dry powder.

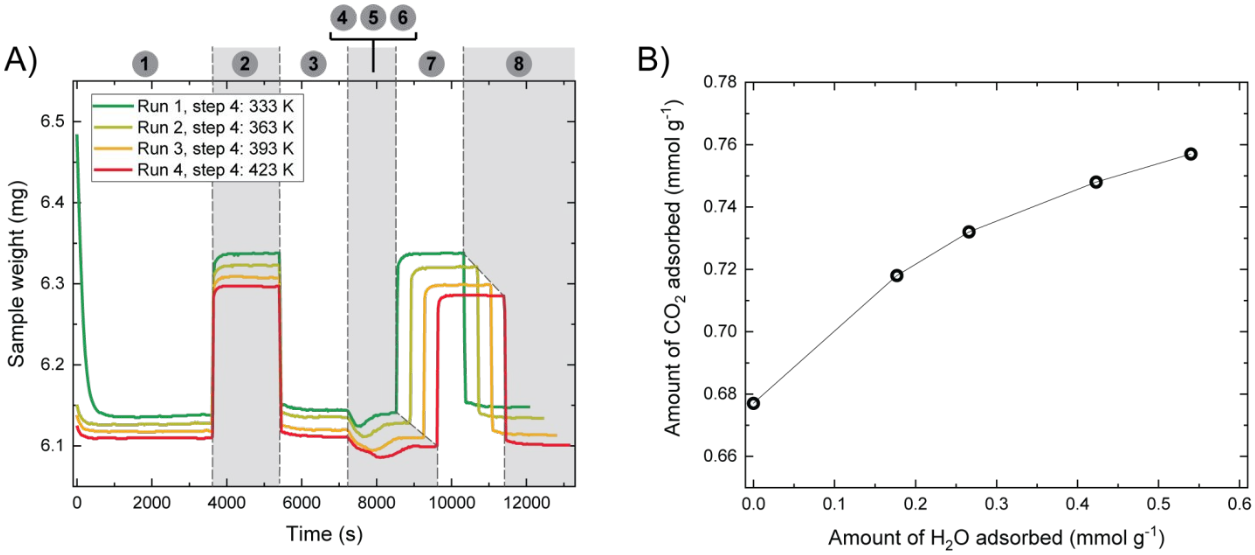

Figure S14. A) TGA curves of water-equilibrated TAPB-NDA-COF, using for every consecutive run the same steps: N_2 flow at 298 K (1), CO_2 flow at 298 K (2), N_2 flow at 298 K (3), heating to indicated temperature and cooling to 298 K under N_2 flow and both rates 10 K min^{-1} (4), CO_2 flow at 298 K (5), N_2 flow at 298 K (6). Flow rates of N_2 and CO_2 were 100 mL·min^{-1}. The pressure is kept constant at atmospheric pressure. **B)** Evolution of CO_2 uptake as a function of the amount of pre-adsorbed water of TAPB-NDA-COF. The line between the data points functions to guide the eye.

Table S2. Comparison of CO_2 capture performance (dry and humid) of TAPB-NDA-COF with other adsorbents, all at 1 bar CO_2 partial pressure.

Adsorbent	BET surface area (m^2 g^{-1})	CO_2 capacity at dry conditions (wt. %)	CO_2 capacity at humid conditions (wt. %)	Reference
TAPB-NDA-COF	722	3.0 at 298 K	3.4 at 298 K and 9 % RH	This work
InOF-1 [$In_2(OH)_2$(BPTC)]	1060	5.4 at 303 K	11 at 303 K and 20 % RH	S2
NOTT-400 [$Sc_2(OH)_2$(BPTC)]	1356	4.2 at 303 K	10.2 at 303 K and 20% RH	S3
NOTT-401 [Sc(OH)(TDA)]	1504	1.2 at 303 K	3.9 at 303 K and 5 % RH	S4
MIL-53(Al) [Al(OH)(BDC)]	1096	3.5 at 303 K	5.2 at 303 K and 20 % RH	S5
MIL-53(Al) [Al(OH)(BDC)]	1096	3.5 at 303 K	6.0 at 303 K and 5 % RH	S6
NH_2-MIL-53(Al) [Al(OH)(NH_2-BDC)]	780	4.9 at 303 K	4.6 at 303 K and 5 % RH	S6
PCN-250(Fe_3)	1470	5.2 at 298 K	8.0 at 298 K and 50 % RH	S7
PCN-250(Fe_2Co)	1653	5.8 at 298 K	9.8 at 298 K and 50 % RH	S7
Cu-BTC (HKUST-1)	Not mentioned (Langmuir surface area of 1492 m^2/g)	22 at 298 K	36 at 298 K and 4 wt% hydrated	S8

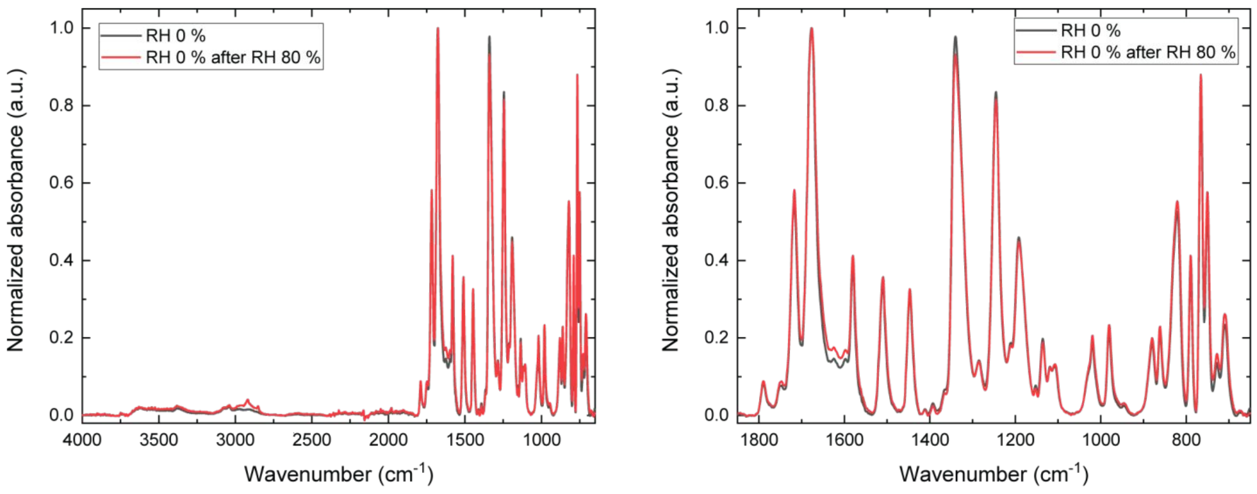

Figure S15. FT-IR spectra of dry TAPB-NDA-COF (black) and dried (degas in vacuum oven at 130 °C overnight) TAPB-NDA-COF after being equilibrated at 298 K and 80 % RH for 16 hours (red). The right graph is a zoom-in of the left graph.

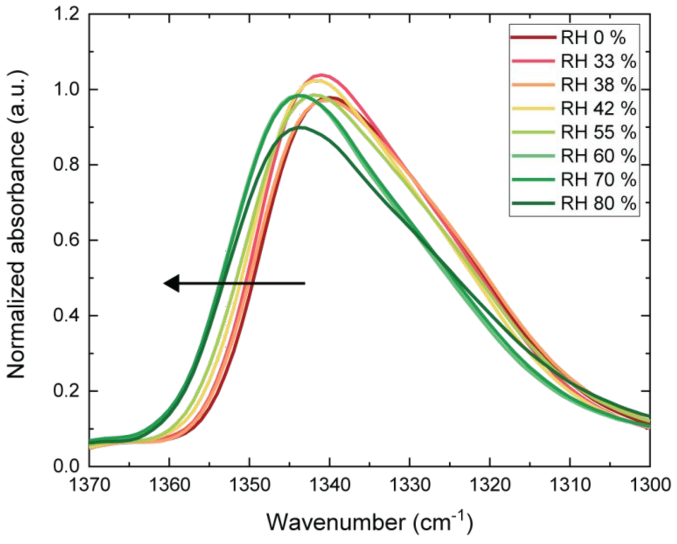

Figure S16. FT-IR spectra of TAPB-NDA-COF equilibrated at 298 K and various RH values, zoom-in on the imide C-N stretch vibration.

References

Ref S1 J. W. M. Osterrieth et al. *Adv.Mater.* **2022**, *34*, 2201502.

Ref S2 R. A. Peralta, B. Alcántar-Vázquez, M. Sánchez-Serratos, E. González-Zamora and I. A. Ibarra, *Inorg. Chem. Front.*, **2015**, *2*, 898–903.

Ref S3 J. R. Álvarez, R. A. Peralta, J. Balmaseda, E. González-Zamora and I. A. Ibarra, *Inorg. Chem. Front.*, **2015**, *2*, 1080–1084.

Ref S4 E. Sánchez-González, J. R. Álvarez, R. A. Peralta, A. Campos-Reales-Pineda, A. Tejeda-Cruz, E. Lima, J. Balmaseda, E. González-Zamora and I. A. Ibarra, *ACS Omega*, **2016**, *1*, 305–310.

Ref S5 M. Sánchez-Serratos, P. A. Bayliss, R. A. Peralta, E. González-Zamora, E. Lima and I. A. Ibarra, *New J. Chem.*, **2016**, *40*, 68–72.

Ref S6 A. Zárate, R. A. Peralta, P. A. Bayliss, R. Howie, M. Sánchez-Serratos, P. Carmona-Monroy, D. Solis-Ibarra, E. González-Zamora and I. A. Ibarra, *RSC Adv.*, **2016**, *6*, 9978–9983.

Ref S7 Chen, Y.; Qiao, Z.; Huang, J.; Wu, H.; Xiao, J.; Xia, Q.; Xi, H.; Hu, J.; Zhou, J.; Li, Z. *ACS Appl. Mater. Interfaces* **2018**, *10*, 38638–38647.

Ref S8 Yazaydin, A. Ö.; Benin, A. I.; Faheem, S. A.; Jakubczak, P.; Low, J. J.; Willis, R. R.; Snurr, R. Q. *Chem. Mater.* **2009**, *21*, 1425–1430.
